# Report of the Society of Biology

**Published:** 1879-04

**Authors:** O. C. Oliver

**Affiliations:** Hotel D’Orient, Paris


### ARTICLE XII.

REPORT OF THE SOCIETY OF BIOLOGY. Meeting of February 1. President M. Paul Bert.

It has been demonstrated in physics that a body cannot be transparent except all its elements (i. e. physical) have the same *index* of refraction. M. Ranvier stated that this was also true of the cornea, composed as it is of divers histological elements. The speaker had investigated the physical conditions which destroy the transparency of the cornea. The cornea of a frog having been excised and fixed was exposed for 5 minutes to the vapors of osmic acid. Examination immediately after by the microscope, the cornea still remaining fixed, failed to reveal a single cell. If, however, the cornea had been plunged in aqueous humor previous to exposing it to the fumes of the osmic acid the corpuscles were at once apparent. From this double experiment the speaker concluded that the corpuscles of the *living* cornea are *invisible*. Under the influence of watery vapor the fibres of the connective tissue swell, thus diminishing their refractive power. Hence results a loss of transparency—due solely to the imbibition.

*Alteration of the Anterior Roots in Saturnine Paralysis.*—M. Déjerine said the authors of late years state that in cases of saturnine paralysis changes in the muscular nerves are constantly met with. (Lancereaux, Gombault, Westphal, Mayor, Friedlaender). This alteration, analogous to that observed in the peripheric end of a cut nerve (parenchymatous neuritis of some authors) is the more marked, the more advanced as a lesion, the longer the duration of the paralysis and muscular atrophy. The same alterations are met with in the radial nerve, but these authors fail to state to what *height* they have found these alterations to extend along the course of the nerve. M. Vulpian, in a case of saturnine paralysis dead in his service, has clearly demonstrated alterations of a certain number of the anterior roots in the cervical region. In this case he very clearly demonstrated the existence of "interstitial neuritis" seated in a certain number of the nerve tubes, some of which were atrophied and reduced to their "sheath of Schwan." The case observed by Prof. Vulpian is the only one up to the present in which examination of the anterior roots has given positive results. M. Déjerine having had occasion to examine the nervous centers and muscles in five cases of saturnine paralysis of the extensors gave the results of his researches. These were examined in the recent condition—the intermuscular and radial nerves, together with the anterior roots—with the following results:

*Alteration of Muscles.*—Simple atrophy of the primitive fasiculi with multiplication of their nuclei, no trace of fatty degeneration.

*Alteration of the Intermuscular Nerves.*—Degenerative atrophy of the nerve tubes, granular degeneration of the myeline, multiplication of the nuclei of the protoplasm, destruction of the axis cylinder. This destruction of the nerve tubes is the direct cause of the duration of the paralysis, moreover, it does not affect all the tubes in the same degree; while some are scarcely changed others are reduced to their "sheath of Schwan," others being altered in all degress from the first-mentioned condition to the last. It is, however, rare to meet with a perfectly sound fiber in a preparation.

*Alterations in the Radial Nerve.*—These same alterations have been found in a certain number of fibers of this nerve in the greater part of its length, and in one case, where the conditions of the autopsy permitted—very near its point of emergence from the brachial plexus.

*Alterations in the Anterior Roots.*—Examination in the recent condition by treating with osmic acid and picro-carmine has been made of the anterior roots of four of these cases. In two cases M. Déjerine has very clearly demonstrated the existence of very advanced alterations in a certain number of nerve tubes, alterations in all respects similar to those met with in the intermuscular and radial nerves. In one of these cases, and this is particularly interesting, the patient had paralysis on one side only—the side in which the alteration of the nerve-roots was observed, the other side being absolutely normal. In the three other cases examined by the same methods, the results were somewhat doubtful as to the alteration of nerve tubes, but they did not present the healthy appearance observed upon the sound side, yet it was impossible to establish any distinct alteration beyond a slight atrophy—not very pronounced. The speaker stated that he should examine the spinal cord in these cases and report at a subsequent meeting. M. Déjerine adding these two cases to that observed by Prof. Vulpian, and considering the clinical history of the affection and, moreover, the fact that it is generally bilateral, offered the hypothesis—under all reservations—that saturnine paralysis is probably of medullary origin, a hypothesis that subsequent researches would either confirm or refute, but which, in the actual state of the science, is the only one we have any rational warrant for, in view of the clinical history and anatomical lesions of this affection. Dr. Charcot observed that he had never found any well-marked changes in the spinal cord in cases of saturnine paralysis. M. Ranvier said he did not comprehend degenerative lesions of the nerves proceeding from the periphery to the center. In these cases of saturnine paralysis the examination of sections never showed him any marked lesion, indeed, so far as he could see, they presented very much the appearance of healthy nerves. M. Déjerine reminded those present of what occurred in stumps of amputations, as surgeons well knew there ensued in the cut nerve an interstitial neuritis, which ascended some centimeters along its length, and yet in these cases there were not (intrinsically) any symptoms of spinal irritation.

*Contraction in Lesions of the Lateral Ventricles.*—M. Cassy gave the results of his experiments upon dogs in the laboratory of Prof. Vulpian. He introduced into the lateral ventricles of a dog (through the corpus callosum) crystals of nitrate of silver; thus producing superficial lesions of the ventricular walls, with slight effusion. These lesions developed little by little, have not given rise, in some carefully observed cases, to any contraction or any convulsive phenomena. Yet contraction is regarded as the rule in effusions into the lateral ventricles. M. Cassy then asks whether the contraction is not due to the pressure upon the neighboring parts of the ventricular walls, distended by the rapid effusion into these cavities, instead of being a phenomon of reflex action due to irritation of their walls by the pressure of the effused material as regarded by the authors.

In support of his hypothesis, the speaker has injected into the lateral ventricles coagulable liquids, boiled starch in one case, and liquified parafine in the other, and he had then seen a contraction intense and general, at the moment of rapid injection of a quantity of liquid relatively small. Without wishing to generalize unwarrantably, or magnify the importance of his observation, M. Cassy believed, in a general way, that the contraction observed in rapid effusions into the lateral ventricles was caused by pressure upon the neighboring parts.

O. C. OLIVER.

HOTEL D'ORIENT, PARIS, Feb. 25, 1879.

